# Use of embedded strain gages for the in-vitro study of proximal tibial cancellous bone deformation during knee flexion-extension movement: development, reproducibility and preliminary results of feasibility after frontal low femoral osteotomy

**DOI:** 10.1186/1749-799X-6-12

**Published:** 2011-03-03

**Authors:** Stéphane Sobczak, Patrick Salvia, Pierre-Michel Dugailly, Philippe Lefèvre, Véronique Feipel, Serge Van Sint Jan, Marcel Rooze

**Affiliations:** 1 Laboratory of Anatomy, Biomechanics and Organogenesis (LABO) (CP 619), Faculty of Medicine, Université Libre de Bruxelles (ULB), Bruxelles, Belgium; 2 Laboratory for Functional Anatomy, Institute for Motor Sciences, Université Libre de Bruxelles (ULB), Bruxelles, Belgium; 3 Department of Orthopedics and Traumatology, Cliniques Universitaires de Bruxelles, Hôpital Erasme, Brussels, Belgium

## Abstract

**Background:**

This paper reports the development of an in-vitro technique allowing quantification of relative (not absolute) deformations measured at the level of the cancellous bone of the tibial proximal epiphysis (CB_TPE_) during knee flexion-extension. This method has been developed to allow a future study of the effects of low femoral osteotomies consequence on the CB_TPE_.

**Methods:**

Six strain gages were encapsulated in an epoxy resin solution to form, after resin polymerisation, six measurement elements (ME). The latter were inserted into the CB_TPE _of six unembalmed specimens, just below the tibial plateau. Knee motion data were collected by three-dimensional (3D) electrogoniometry during several cycles of knee flexion-extension. Intra- and inter-observer reproducibility was estimated on one specimen for all MEs. Intra-specimen repeatability was calculated to determine specimen's variability and the error of measurement. A varum and valgum chirurgical procedure was realised on another specimen to observed CB_TPE _deformation after these kind of procedure.

**Results:**

Average intra-observer variation of the deformation ranged from 8% to 9% (mean coefficient of variation, MCV) respectively for extension and flexion movement. The coefficient of multiple correlations (CMC) ranged from 0.93 to 0.96 for flexion and extension. No phase shift of maximum strain peaks was observed. Inter-observer MCV averaged 23% and 28% for flexion and extension. The CMC were 0.82 and 0.87 respectively for extension and flexion. For the intra-specimen repeatability, the average of mean RMS difference and the mean ICC were calculated only for flexion movement. The mean RMS variability ranged from 7 to 10% and the mean ICC was 0.98 (0.95 - 0.99). A Pearson's correlation coefficient was calculated showing that RMS was independent of signal intensity. For the chirurgical procedure, valgum and varum deviation seems be in agree with the frontal misalignment theory.

**Conclusions:**

Results show that the methodology is reproducible within a range of 10%. This method has been developed to allow analysis the indirect reflect of deformation variations in CB_TPE _before and after distal femoral osteotomies. The first results of the valgum and varum deformation show that our methodology allows this kind of measurement and are encourageant for latter studies. It will therefore allow quantification and enhance the understanding of the effects of this kind of surgery on the CB_TPE _loading.

## Background

Valgus deformity of the knee is a well-known factor in the aetiology of lateralized gonarthrosis. Following Pauwel's theory [1], Maquet proposed to use frontal lower limb realignment techniques [2-4]. However, in 25 to 30% of the cases, pain persists after treatment [5]. For other authors [6], 3D correction of knee joint deformation could improve the outcome of the realignment treatment. Only one experimental study on the effects of high tibial osteotomies on knee joint kinematics and muscular moment arms of thigh muscles has been published [7]. Several studies [8-11] reported joint constraint distribution and contact area distribution for various joints, various static positions and loading conditions. The methods utilised do not allow tracking of in-vivo femoro-tibial articular deformations during continuous knee flexion-extension motion despite the usefulness of these studies. In-vitro and in-vivo analysis of dynamic joint deformation patterns is also still a challenge. The mechanical stress in bones cannot be measured in a living subject without the use of invasive surgical procedures, due to obvious ethical concerns. Indirect alternatives can be found in finite element modelling generated from medical imaging [12]. Previous studies [13-15] reported results on deformations of the cancellous bone of the tibial proximal epiphysis (CB_TPE_) in static conditions evaluating the mechanical characteristics of different extracted bone portion in compression, traction and/or torsion. This paper proposes an in-vitro method to study the CB_TPE _deformations using embedded strain gages during continuous knee motions. Embedded strain gages were used previously by some authors to measure hip prosthesis cement deformation [16] or investigate the stress in the cement layer underlying tibial plateau [17]. The utilisation of embedded strain gages is complex and difficult.

This study did not aim at analyzing the force transmission in the femoro-tibial joint compartment and did not allow absolute CB_TPE_. The aim was the quantification the indirect reflect in the CB_TPE _of tibial plateau loading reported by the presented measurement method before and after various kinds of osteotomies. With such data, it will be possible to analyze the relative variations of the local deformation and to increase our understanding of the relationship between constraint patterns and overall joint motion. Indeed, the real 3D impact of osteotomies on tibial plateau loading is poorly reported in the literature [7]. The direct method presented here should provide innovative data on that particular topic.

## Methods

### Specimens and setting

**Figure 1:**
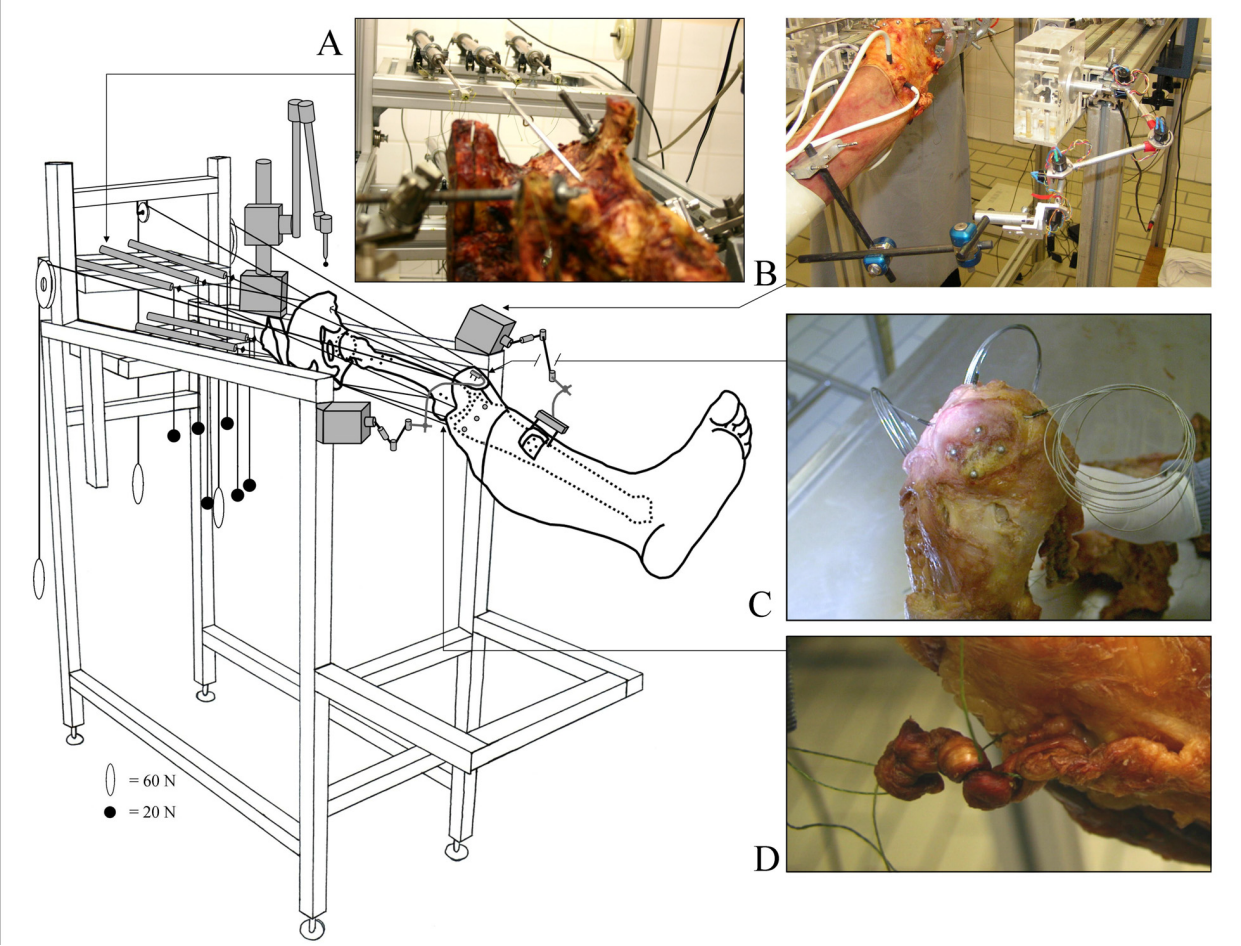
**Schematic view of the experimental setting**. Schematic view of the experimental setting showing a specimen mounted rigidly on the experimental jig in anatomical position. A: representation of the LVDT placed in the prolongation of the action line of different muscles. B: tibial 3D electrogoniometer. C: illustration of the loading muscles, RF, VL and VM by cerclage with metallic wires. D: representation of the fixation of the other muscles according to the bull's method.

Six fresh-frozen lower limbs (average age: 84 ± 9 years; 4 males, 2 females) were obtained from the ULB Body Donation program. Thawing occurred at room temperature 24 hours before specimen preparation and experiment. Each specimen included a full lower limb with its hemi-pelvis. The pelvis and femur were rigidly mounted on the experimental jig in an anatomical position (Figure 1). The distal tendon of 8 muscles of interest was carefully dissected and cut at their distal musculoskeletal junction. Distal muscle attachments were left intact. Muscles dissected were the rectus femoris (RF), vastus lateralis (VL), vastus intermedius (VI), vastus medialis (VM), biceps femoris (BF), semitendinosus (ST), semimembranosus (SM), gracilis (Grac) and tensor fasciae latae (TFL). All other muscles were kept intact. Special care was given to respect the integrity of hip and knee joint capsule and ligaments. One fishing wire (Surflon^®^, Nylon coated, American Fishing Wire, 90 Lb., USA) was attached to each dissected tendon (Figure 1D) by an orthopaedic surgeon according to Bull's method [18,19]. Each fishing wire ran proximally through tunnels drilled into the bone at the level of the related muscle origin to allow joint loading following the physiological muscle lines of action. Total loading was 300 N (RF + VM = 80 N; VL and VI = 60 N each; BF, ST, SM, Grac, and TFL = 20 N each). Muscle loading was selected to respect the forces that each muscle could generate; this was determined from muscle volume and muscle pennation angle [20]. The fishing wire of selected muscles (RF, BF, ST, SM, Grac and TFL) were attached to the mobile axis of six Linear Variable Displacement Transductors (LVDT, Solartron Metrology^®^, West Sussex, UK) to measure tendon excursion during flexion-extension movement (Figure 1A). Two electrogoniometers [21-23] were used to collect continuous femoro-patellar and femoro-tibial 3D kinematics (Figure 1B). The results of tendon excursion and full joint kinematics are not presented here.

### Measurement Element (ME)

**Figure 2:**
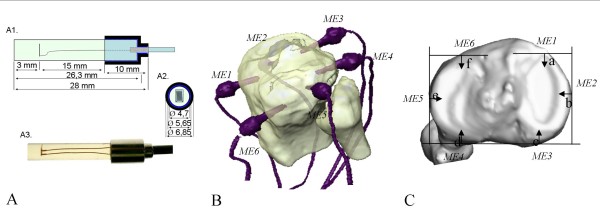
**Representation and location of measurement element**. Schematic view and location of MEs. A1 and A2: Schematic view of the ME. A3: ME used in this study including a Strain Gage. B and C: 3D representation (obtained from medical imaging) of ME locations in the proximal epiphysis of the tibia. ME1 and ME6: most anterior edge of the medial and lateral condyles, respectively; ME2: most medial point of the medial condyle, ME3 and ME4 = most posterior edge of the medial and lateral condyles, respectively. M5: most lateral point of the lateral condyle (tunnels were drilled 10 mm below these landmarks, see text for further details).

Six MEs were used in this study. Each ME included two components: a strain gage (SG) and an epoxy resin cylinder. The strain gage was made from cupronickel alloy (60/40) (Rosette unidirectional^®^, FLA-1-17, length: 3 mm, 120 Ω, TML, Tokyo, Japan) and was embedded in an epoxy resin solution (LX 112^®^, Ladd Research Industries, Williston, USA) (Figure 2A). The resin was selected for its low shrinkage properties during polymerisation to avoid damage of the SG. Small polypropylene tubes (Ø: 4.7 mm) were used as moulds to encapsulate the SG in the resin. After polymerisation (1 day at 30°C and 1 day at 60°C), the MEs were cooled down at room temperature.

### Determination of epoxy resin Young's modulus

Young's modulus of the epoxy resin was determined to define the influence of the epoxy resin on further measurement. Ten resin cylinders (L: 12 mm, H: 30 mm) were processed. Cylinders were placed in a servohydraulic test system (MTS 858 Mini Bionix, 10-kN loading head, Eden Prairie, Minnesota, USA). Compression force was applied along the longitudinal axis of the cylinders and was released at the yield point (i.e., corresponding to the limit of the cylinder elastic range). Young's modulus was then calculated in the elastic part of the cylinder deformation [24]. The epoxy resin Young's modulus (*E*) was obtained by equation 1. The average value was 2.09 ± 0. 03 GPa.

(1) $$E=\frac{\left(F/S\right)}{\left(\Delta L/L0\right)}$$

Where:

E = Young's modulus (Pa)

F = Force applied (N).

S = cross-sectional area of cylinder (m2).

ΔL = Length variation (m).

L0 = Initial length (m).

### Data acquisition

A custom-made device including six amplifying modules was developed to collect the output from the 6 SGs (Figure 2B). Each module included a Wheatstone bridge which allowed the measurement of tension imbalance in the bridge. These modules were supplied in differential current (DC) with a floating power supply of 9V. Each Wheatstone bridge was adjusted using an offset correction and the parasite noise related to external interferences were eliminated (common mode rejection). The ratio between the signal power and the parasite noise power (i.e., S/N ratio) was equal to 66 db. All modules were connected to an acquisition board (DAP3200a, Microstar Laboratories). The acquisition frequency was 208 Hz.

### Definition of the strain gage deformation

The deformation (ε) was obtained by equation 2 [25]. This equation shows the relation between the resistance variation (ΔR/R0) and the gage factor (G_F_). The gage factor defines the SG deformation in a well-defined direction. G_F _was given by the gage manufacturer and was equal to 2.15 ± 1%. This value was constant and independent from the load applied.

(2) $$\varepsilon =\frac{\Delta R/Ro}{G_{F}}$$

Where:

ε = Deformation

ΔR = Resistance variation.

Ro = Nominal resistance of SG (120Ω).

G_F _= Gage factor

### Calibration

A custom-made device was built to measure the SG length variation (ΔL) and to perform SG calibration. A SG was embedded into a LX 112 epoxy resin plate (L = 30 mm, l = 12 mm, d = 3 mm) linked to a mechanism (Figure 2E). The latter mechanism allowed stretching of both resin plate and SG in a controlled way. The elongation range was from 1 μm to 50 μm. Deformation results were expressed in resistance variation to obtain a calibration equation (Eq. 3) after Analog-to-Digital Conversion (ADC). The final deformation ε was given by equation 4. Note that this calibration was dependent on the above-determined epoxy resin Young's modulus (see Eq. 1).

(3) $$f\Delta r(x)=(3^{e-6}x-0.0002)$$

(4) $$\varepsilon =\frac{(3^{e-6}x-0.0002)/Ro}{G_{F}}$$

Where:

ε = Deformation

x = ADC variations

Ro = Nominal resistance of SG (120Ω).

G_F _= Gage factor

### Location of MEs

ME location was standardized using computed tomography (CT, Siemens SOMATRON, helical mode, slice thickness = 0.5 mm, inter-slice spacing = 0.5 mm, image format = DICOM 2.0) on each specimen before constraint experiments took place. CT data segmentation and 3D-model reconstruction of bones were performed using a dedicated software interface (Amira^®^, Visage Imaging, Inc., San Diego, USA). Six tunnels (Ø: 4.2 mm) were drilled into the cancellous bone underlying the tibial plateau at 10 mm below the joint line. Tunnel depths were 13 mm and strain gauges were placed 10 mm from the cortical bone (Figure 2 A1). ME locations were standardized using strict definitions describing anatomical landmark locations on the available 3D models (Figure 2C). Tunnel orientation was parallel to the cartilaginous surface of the tibia. Tunnel diameter size was slightly smaller than the ME diameter to ensure a tight fit with and a maximal contact between the MEs and the cancellous bone. One ME was introduced into each tunnel (Figure 2D), and no glue was used.

### Experimental protocol

For each specimen, three repetitions of two cycles of flexion-extension movement were performed. Measurement started with the specimen knee in full extension maintained by the muscle loading. Flexion was then performed manually by pushing with an open hand on the distal part of anterior face of the leg. Once knee flexion was obtained, the manual pressure was released and the knee passively moved back by the muscle loading of the quadricipital tendon. All above-mentioned muscle loading was kept in place during the entire measurement session. Intra- and inter-observer reproducibility was analysed on one specimen (specimen 1). To allow reproducibility analysis, three operators independently performed the above data collection (three trials of three repetitions including two motion cycles) on the same specimen within a three-hour interval. Valgum and Varum procedure were realised on another specimen. These angular corrections (6° and 12°) were applied using a specially-built control system. Before processing each repetition for further analysis, the extension of the first cycle and the flexion of the second cycle were selected to avoid experimental noise (i.e., system oscillations) usually observable at the beginning and end of movement. Data were then normalized to flexion-extension range of motion.

### Statistical analysis

Inter- and intra-observer reproducibility was analyzed by coefficients of multiple correlation (CMC) and mean coefficient of variation (MCV). Intra-observer reproducibility was obtained by comparing the mean of the 3 repetitions of the three trials for one operator. Inter-observer reproducibility was performed by comparison of the mean of the 3 repetitions performed by each operator at 3 different times. For intra-specimen repeatability we calculated the mean RMS difference between the three repetitions for each ME. An ICC (3.1 Two-Way Mixed) was also calculated. These were then averaged across MEs and subjects. Pearson's correlation coefficients were calculated to analyse if the RMS was dependent of data amplitude during movement. For valgum and varum deviations comparison, a faithful analysis of the different patterns of curve was carried out.

## Results

### Intra-observer reproducibility

**Figure 3:**
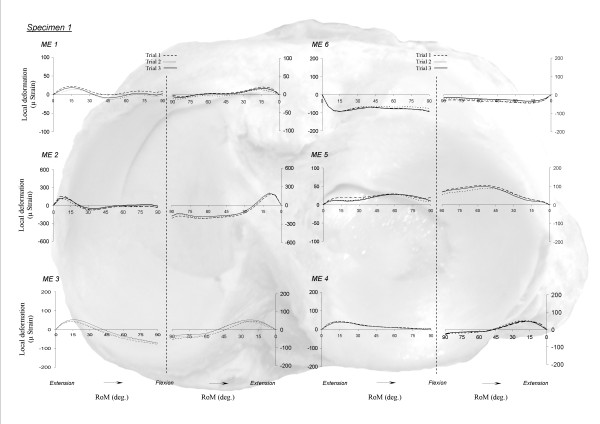
**Intra-operator reproducibility**. Intra-operator variability during flexion and extension motion. ME 1: antero-medial strain gage; ME 2: medial strain gage; ME 3: postero-medial strain gage; ME 4: postero-lateral strain gage; ME 5: lateral strain gage; ME 6: antero-lateral strain gage.

Good superposition of different repetitions was obtained for all MEs (Figure 3). The CMC showed a mean value of 0.93 for flexion (range: 0.83-0.99) and of 0.96 for extension (range: 0.96-0.99). Mean MCV values were obtained during knee extension (8%, range: 4-13%) and flexion (9%, range: 6- 12%) (Table 1). Deformation patterns were similar between repetitions performed by the same operator.

**Table 1:** Intra- and inter-observer reliability

	*Extension to flexion*					*Flexion to extension*			
	**Intra-observer reliability**		**Inter-observer reliability**			**Intra-observer reliability**		**Inter-observer reliability**	

	*MCV(%)*	*CMC*	*MCV(%)*	*CMC*		*MCV(%)*	*CMC*	*MCV(%)*	*CMC*

**ME1**	12	0.83	17	0.97	**ME1**	6	0.96	15	0.99

**ME2**	11	0.98	12	0.95	**ME2**	9	0.97	21	0.93

**ME3**	9	0.96	13	0.98	**ME3**	7	0.97	16	0.97

**ME4**	6	0.99	17	0.88	**ME4**	4	0.99	28	0.63

**ME5**	8	0.92	43	0.70	**ME5**	13	0.88	33	0.73

**ME6**	10	0.88	34	0.76	**ME6**	7	0.98	55	0.65

***Mean***	***9***	***0.93***	***23***	***0.87***	***Mean***	***8***	***0.96***	***28***	***0.82***

Inter- and intra-observer mean coefficient of variation (in %) and coefficient of multiple correlation. ME1: antero-medial Strain gage; ME2: medial Strain gage; ME3: postero-medial Strain gage; ME4: postero-lateral Strain gage; ME5: lateral Strain gage; ME6: antero-lateral Strain gage.

### Inter-observer reproducibility

**Figure 4:**
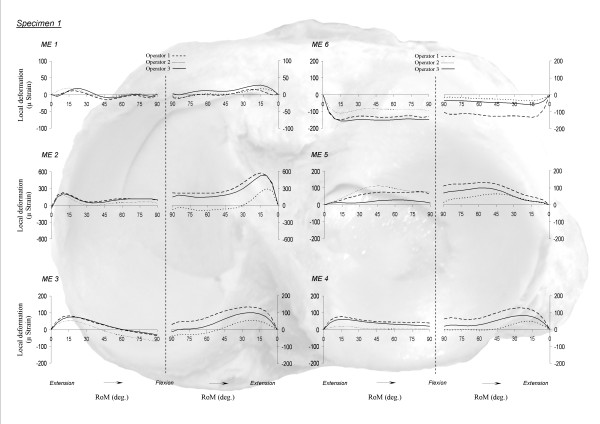
**Inter-operator reproducibility**. Inter-operator variability during flexion and extension motion. ME 1: antero-medial strain gage; ME 2: medial strain gage; ME 3: postero-medial strain gage; ME 4: postero-lateral strain gage; ME 5: lateral strain gage; ME 6: antero-lateral strain gage.

MCV for all MEs averaged 23% (range: 12-43%) and 28% (range: 15-55%) during flexion and extension, respectively. Mean CMC were 0.87 (range: 0.70-0.98) and 0.82 (range: 0.63-0.99) (Table 1). The deformation magnitudes (Figure 4) differed between operators, but the shape of the curves showed similar deformation patterns.

### Intra-specimen repeatability

**Figure 5:**
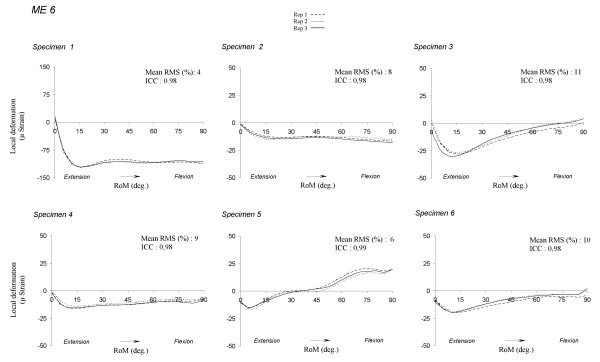
**Deformation repeatability for ME 6**. Repeatability measurement for all specimens to the ME 6. ME 1: antero-medial strain gage; ME 2: medial strain gage; ME 3: postero-medial strain gage; ME 4: postero-lateral strain gage; ME 5: lateral strain gage; ME 6: antero-lateral strain gage.

As intra-observer reproducibility data was not statistically different, this analysis was carried out for the flexion movement. Figure 5 presents graphically the phenomenon on ME6. For all MEs and specimens, the mean RMS differences (%) ranged from 3 to 15% and the mean ICC ranged from 0.95 to 0.99 (Table 2). The average mean RMS difference of the sample ranged from 7 and 10%. The mean correlation coefficient was ranged from -0.22 and 0.55. These values imply that the RMS differences were independent of signal intensity.

**Table 2:** Intra-specimen repeatability

	*Mean Range*							*Mean Range*							*Mean Range*						
**Specimen**	Max (mS)	Min (mS)	Mean RMS (mS)	Mean RMS (%)	ICC		r	Max (mS)	Min (mS)	Mean RMS (mS)	Mean RMS (%)	ICC		r	Max (mS)	Min (mS)	Mean RMS (mS)	Mean RMS (%)	ICC		r

	ME1							ME2							ME3						

**S1**	13	-10	2	7	0.98	*	0.10	288	27	28	9	0.98	*	-0.35	71	-44	3	3	0.99	*	0.13

**S2**	7	-11	2	12	0.95	*	-0.35	96	-6	10	10	0.99	*	-0,20	162	-19	18	10	0.97	*	0.27

**S3**	-14	-49	3	5	0.98	*	0.36	72	-147	25	11	0.99	*	0.59	35	6	3	6	0.99	*	-0.20

**S4**	14	-18	2	5	0.97	*	0.23	33	-62	6	6	0.98	*	0.65	77	-4	8	10	0.99	*	0.41

**S5**	35	-68	5	5	0.99	*	0.78	-3	-26	1	5	0.99	*	0.32	15	-1	1	9	0.98	*	-0.16

**S6**	-3	-39	4	9	0.98	*	-0.46	148	20	10	6	0.95	*	0.07	40	-4	4	10	0.98	*	0.04

***Average***	***9***	***-33***	***3***	***7***	***0.98***		***0.11***	***106***	***-32***	***13***	***8***	***0.98***		***0.41***	***67***	***-11***	***6***	***8***	***0.98***		***0.08***

	ME4							ME5							ME6						

**S1**	74	5	3	4	0.99	*	-0.54	36	-3	5	13	0.99	*	-0.60	10	-121	4	3	0.98	*	0.70

**S2**	23	-81	9	8	0.99	*	-0.06	-2	-45	5	11	0.97	*	0.53	-1	-16	1	8	0.98	*	0.49

**S3**	11	-46	7	12	0.99	*	0.42	-4	-33	4	11	0.95	*	0.74	4	-28	3	11	0.98	*	0.45

**S4**	29	-42	11	15	0.98	*	-0.68	-3	-10	1	5	0.97	*	0.38	-1	-15	1	9	0.98	*	0.55

**S5**	-4	-57	5	7	0.98	*	-0.76	23	-24	5	10	0.99	*	0.43	19	-15	2	6	0.99	*	0.60

**S6**	0	-49	4	9	0.96	*	0.30	52	-14	6	10	0.98	*	0.30	-1	-19	2	10	0.98	*	0.49

***Average***	***22***	***-45***	***6***	***9***	***0.98***	***-0.22***		***17***	***-21***	***4***	***10***	***0.98***		***0.48***	***5***	***-36***	***2***	***8***	***0.98***		***0.55***

Intra-specimen repeatability for all MEs in flexion. ME1: antero-medial Strain gage; ME2: medial Strain gage; ME3: postero-medial Strain gage; ME4: postero-lateral Strain gage; ME5: lateral Strain gage; ME6: antero-lateral Strain gage. *:P < 0.0001.

### Frontal distal femoral osteotomy

**Figure 6:**
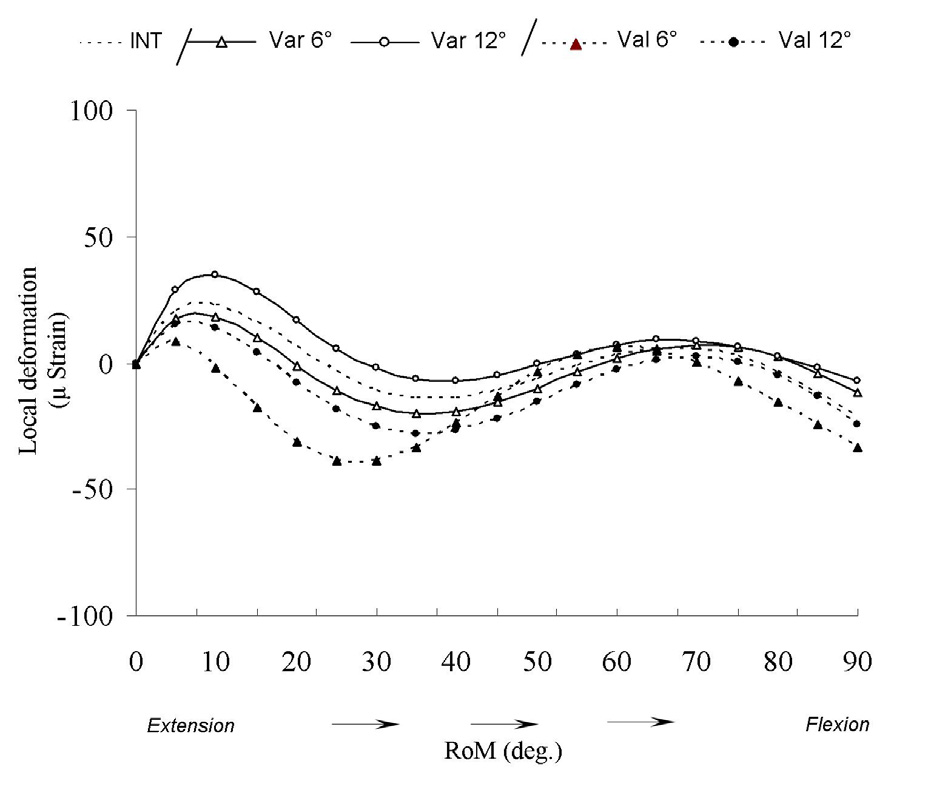
**Valgum and varum deviation on the cancellous bone deformation for ME 2**. Representation of cancellous bone deformation for ME2 after varum and valgum procedure during knee flexion. INT: intact; Var 6°: varum 6°; Var 12°: varum 12°; Val 6°: valgum 6°; Val 12°: valgum 12°.

Figure 6 shows after varum and valgum deviation the cancellous bone deformation variability of the medial measurement element (ME2). We could divide the flexion motion in three phases: the first from 0° until 30°, the second from 30° until 65° and the third from 65° until 90°. During the first phase, the valgum deviations induce a decrease of CB_TPE _compared to the intact condition. These decreases were not proportional of the degrees of deviation. For varum deviations, only the condition of 12° increases the CB_TPE_. During the second phase, the curve patterns of valgum 6° condition increase until similar values of varum 12° condition. During the third phase, the varum deformations increase the CB_TPE _compared to the intact condition and inversely for valgum deformations.

## Discussion

These results show that the developed method allows reproducible in-vitro measurements of the indirect reflect of deformation variations occurring in the CB_TPE _during knee flexion-extension. We are conscious that the strain gages are designed for being bonded onto the surface of structure but previous study [16,17] used strain gages into structure. The cancellous bone is not homogenous and anisotropic and the orientation and location of the trabecula are very important to loading transfer. The strain field in structure point is three dimensional. There are three normal strains and three shear strains. In our study we decided consider the measurement of vertical strain. Note that data was dependent of the epoxy resin deformation. The data obtained were the indirect reflect of the cancellous bone load transfer. Nevertheless, the introduction of different structure in the cancellous bone could create a local reinforcement and modify the cancellous bone mechanics. During dynamics of gait, ground reaction force is of primary importance to explain joint loading. In our experimental setting, an open kinematic chain was studied. It would be therefore interesting to reproduce this study in a closed kinematic chain setting to take into account the contribution of ground reaction force, that might affect cancellous bone deformation differently as compared to loading along muscle lines of actions.

Intra-observer reproducibility was satisfactory for both MCV (not exceeding 10%) and CMC (above 0.93). Inter-observer reproducibility indicated that similar measured deformation patterns could be found for all operators at all ME locations (CMC mean: 0.82 to 0.87), although these patterns showed different ranges. It could have been advanced that this range difference could be related to the different velocities applied by the operator to flex the knee joint. We studied the correlation between the mean RMS values and mean and maximal primary motion velocities. The coefficients of determination (r^2^) between RMS and motion velocity and were 0.32 and 0.39, respectively for mean and maximal velocities. These results do thus not support that velocity was a factor influencing local bone deformation.

For intra-specimen repeatability we chose to analyse the flexion movement. Indeed, no difference between flexion and extension intra-observer reproducibility was observed. Moreover, it seems more logical to express our data according to flexion movement. Indeed, knee joint kinematics and muscular moment arms that are pertinent to interpret deformation data are generally expressed during this movement. The average mean RMS differences (7 to 10%) and the Mean ICC (0.95 to 0.99) showed that maximum variability did not exceed 10% and that a great similarity of the curves was observed. The mean correlation coefficient was ranged from -0.22 and 0.55, indicating that RMS differences were independent of the signal intensity. This implies that the measurement error is constant and does not exceed 10%

The curve pattern of ME6 in all specimens (Figure 5) suggests individual variability of knee deformations. The intensity variability may be due to some discrepancies in gage placement even if this was standardized, to the quality of cancellous bone, especially in elderly people [13,26]. Indeed, this is approximately 654 (± 304) MPa in young subjects, 829 (± 422) MPa in adults and 613 (± 319) MPa in elderly people [13]. The variability could also be due to individual anatomical and/or kinematical variations (e.g. joint geometry, presence or absence of inconstant ligaments, motion patterns).

Compression tests of the resin cylinders indicated that the average value of the resin Young's modulus was 2.09 (± 0.03) GPa. In comparison to the CB_TPE _Young's modulus [14], the resin Young's modulus is 2.4 to 66 times larger than the CB_TPE_. This means that the epoxy resin deformation is smaller than the cancellous bone's and that the data obtained via the MEs underestimated the real deformation of the cancellous bone. However, this system is satisfactory to answer the main aim of this study, which was developing a method to compare bone deformation variations between two conditions (i.e., before and after osteotomies). For example, Figure 6 showed the CB_TPE _deformation for ME2 after varum and valgum deviation for another specimen. The data showed that the deformation patterns seems to be in agree with the frontal deviation theory [1,2] were varum deformity induce a medial shift of the mechanical axis of the lower limb, increasing medial tibial plateau constraint and inversely for valgum deformity. We showed that before 65°, the varum 6° condition decrease CB_TPE_. After 65° the varum 6° condition increase CB_TPE _compared to the intact condition. The valgum conditions were not proportional to the degree of frontal deformation. This fact could be due to the modification of muscles and ligaments tensions. But this hypothesis should be still confirmed thanks to confrontation with kinematics and moment arm data. Even if the introduction of a rigid element into the cancellous bone can induce a modification of its mechanic behavior, these preliminary results showed that our methodology allows objective measurement of this problematic.

The tunnel size has been selected after several trials to optimize the contact surface between cancellous bone and ME. The MEs were only introduce on CB_TPE _and no glue was used. No sliding and movement of the ME in the tunnel were observed. There is currently no other direct method which allows validation of ME output data. To the authors' knowledge, no other 'direct' method is available to record cancellous bone deformations during motion. Only indirect methods exist [12,13] and these deal with static positions and therefore are not suitable to validate the protocol presented in this study. The latter is the first method which allows to analyze directly in-situ the variations of cancellous bone during a joint movement. The deformation pattern for each individual specimen in some well-defined conditions (e.g., osteotomy) can therefore be compared to each other. Unfortunately, the method does not allow the absolute strain values to be obtained since there is no other direct method is available from the literature for validation.

## Conclusions

The presented method allows the reproducible relative quantification of deformation variations measured in-situ at the cancellous bone of the tibial proximal epiphysis. The method has been used in this paper on the tibial plateau. Intra-observer reproducibility was very good and the measurement error did not exceed 10% in average. Inter-observer reproducibility was less acceptable. Different behaviours were observed among specimens. These were probably due to individual variations in bone quality as previously reported in the literature [13]. The method can now be used for the first time to quantify relative bone deformations before and after distal femoral osteotomies if applied by the same operator. The preliminary results of valgum and varum condition seem in agree with frontal misalignment theory. CB_TPE _deformation measurements could be confronted to knee kinematics analysis and thigh muscular moment arms [7]. This will allow a better understanding of the mechanism of lateralized gonarthrosis, and contribute to the development of more appropriate treatments in the future.

## Abbreviations

CB_TPE_: cancellous bone of the tibial proximal epiphysis; MCV: mean coefficient of variation; CMC: coefficient of multiple correlations, RMS: Root Mean Square; Strain gage: SG: Measure Element: ME; RF: rectus femoris; VL: vastus lateralis; VI: vastus intermedius; VM: vastus medialis; BF: biceps femoris; ST: semitendinosus; SM: semimembranosus; Grac: gracilis; TFL: tensor fasciae latae; LVDT: Linear Variable Displacement Transductors.

## Competing interests

The authors declare that they have no competing interests.

## Authors' contributions

SS: designed the study, carried out the experiments, analysed the result and drafted the manuscript. PS: designed the study, collected and analysed the data. PMD and PL: carried out the experiments. VF, SVSJ and MR: read and approved the final manuscript. All authors read and approved the final manuscript

## References

[B1] Pauwels F Springer-Verlag Biomecanique de l'appareil moteur: contribution à l'étude de l'anatomie fonctionnelle 1979 1 Berlin

[B2] Maquet P The biomechanics of the knee and surgical possibilities of healing osteoarthritic knee joints Clin Orthop Relat Res 1980 149 102 10 7371239

[B3] Edgerton BC Mariani EM Morrey BF Distal femoral varus osteotomy for painful genu valgum: A five-to-11-year follow-up study Clin Orthop Relat Res 1993 288 263 9 8458142

[B4] Li G Papannagari R Most E Park SE Johnson T Tanamal L Rubash HE Anterior tibial post impingement in a posterior stabilized total knee arthroplasty J Orthop Research 2005 23 536 41 10.1016/j.orthres.2004.09.005 15885472

[B5] Aglietti P Menchetti PP Distal femoral varus osteotomy in the valgus osteoarthritic knee Am J Knee Surg 2000 13 89 95 11281336

[B6] Goutallier D Garabedian JM Allain J Bernageau J Influence of lower limb torsionnal deformities on the development of femoro-tibial degenerative arthritis Rev Chir Orthop 1997 83 613 21 9515129

[B7] Baillon B Salvia P Feipel V Rooze M Modifications de la cinématique du genou et des bras de levier du quadriceps et des ischio-jambiers après ostéotomie tibiale haute " curviplane " de valgisation ou de varisation RCO 2006 92 464 72 10.1016/s0035-1040(06)75833-0 17088740

[B8] Coughlin KM Peura GD Fleming BC Hallock S Beynnon BD In vivo loads in the medial compartment of the rabbit knee Clin Biomech (Bristol, Avon) 2005 20 1007 9 10.1016/j.clinbiomech.2005.06.015 16099082

[B9] Fukubayashi T Kurusawa H The contact area and pressure distribution pattern of the knee Acta Orthop Scand 1980 51 871 9 10.3109/17453678008990887 6894212

[B10] Liu ZJ Herring SW Bone surface strains and internal bony pressures at the jaw joint of the miniature pig during masticatory muscle contraction Arch Oral Biol 2000 42 95 112 10.1016/S0003-9969(99)00127-2 10716614

[B11] Wretenberg P Ramsey DK Németh G Tibiofemoral contact points relative to flexion angle measured with MRI Clin Biomech 2002 17 477 85 10.1016/S0268-0033(02)00036-0 12135550

[B12] Taddei F Cristofolini L Martelli S Gill HS Viceconti M Subject-specific finite element models of long bones: An in vitro evaluation of the overall accuracy J. Biomech 2006 39 2457 67 10.1016/j.jbiomech.2005.07.018 16213507

[B13] Ding M Dalstra M Danielsen CC Kabel J Hvid I Linde F Age variations in the properties of human tibial trabecular bone J bone Joint Surg (br) 1997 79-b 995 1002 10.1302/0301-620X.79B6.7538 9393920

[B14] Goldstein SA Wilson DL Sonstegard DA Matthews LS The mechanical properties of human tibial trabecular bone as a function of metaphyseal location J biomech 1983 16 965 69 10.1016/0021-9290(83)90097-0 6671987

[B15] Lancianese SL Kwok E Beck CA Lerner AL Predicting variations in trabecular bone mechanical properties within the human proximal tibia using MR imaging Bone 2008 43 1039 46 10.1016/j.bone.2008.07.247 18755303

[B16] Cristofolini L Viceconti M Development and validation of a technique for strain measurement inside polymethyl methacrylate J Strain Analysis 2000 35 21 33 10.1243/0309324001513982

[B17] Little EG O'Keefe D An experimental technique for the investigation of three-dimensional stress in bone cement underlying a tibial plateau Proc Instn Mech Engrs Part H 1989 203 35 41 10.1243/PIME_PROC_1989_203_005_01 2712951

[B18] Amis AA Oguz C Bull AMJ Senavongse W Dejour D The effect of trochleoplasty on patellar stability and kinematics: A biomechanical study in vitro J bone Joint Surg (br) 2008 90 7 864 9 18591593 10.1302/0301-620X.90B7.20447

[B19] Bull AMJ Berkshire FH Amis AA Accuracy of an electromagnetic measurement device and application to the measurement and description of knee joint motion Proc Instn Mech Engrs Part H 1998 212 347 55 10.1243/0954411981534123 9803154

[B20] Klein Horsman MD Koopman H Van der Helm F Poliacu Prosé L Veeger H Morphological muscle and joint parameters for musculoskeletal modelling of the lower extremity Clin Biomech (Bristol, Avon) 2007 22 239 47 10.1016/j.clinbiomech.2006.10.003 17134801

[B21] Salvia P Développement et application de l'électrogoniométrie tridimensionnelle à l'étude expérimentale et clinique de la cinématique articulaire. PhD thesis 2004 Université Libre de Bruxelles, Department of Anatomy

[B22] Sholukha V Salvia P Hilal I Feipel V Rooze M Van Sint Jan S Calibration and validation of 6 DOFs instrumented spatial linkage for biomechanics applications: A practical approach Med Eng & phys 2004 26 251 60 10.1016/j.medengphy.2003.10.002 14984847

[B23] Van Sint Jan S Salvia P Hilal I Sholukha V Rooze M Clapworthy G Registration of 6-DOFs electrogoniometry and CT medical imaging for 3D joint modelling J Biomech 2002 35 1475 84 10.1016/S0021-9290(02)00074-X 12413966

[B24] Beaupied H Lespessailles E Benhamou CL Evaluation of macrostructural bone biomechanics Rev Rhum 2007 74 447 454 10.1016/j.rhum.2007.02.001 17382570

[B25] Murray WM Miller WR The Bonded Electrical Resistance Strain Gage: an introduction 1992 1 Oxford University Press. New York

[B26] Khodadadyan-Klostermann C Von Seebach M Taylor WR Duda GN Haas NP Distribution of bone mineral density with age and gender in the proximal tibia Clin Biomech 2004 19 370 376 10.1016/j.clinbiomech.2003.12.013 15109757

